# Dolphins, sharks, and barnacles: Use of photographs to examine intra‐ and inter‐specific interactions in bottlenose dolphins in Mozambique

**DOI:** 10.1002/ece3.11691

**Published:** 2024-08-07

**Authors:** Sarah A. Marley, Laura McConnell, Chloe Allen, Shaye Wettner, Thomas Hunt, Diana Rocha, Angie Gullan

**Affiliations:** ^1^ Scotland's Rural College, Craibstone Estate Aberdeen UK; ^2^ Institute of Marine Sciences University of Portsmouth Southsea UK; ^3^ School of the Environment, Geography and Geosciences University of Portsmouth Portsmouth UK; ^4^ Dolphin Encountours Research Center Ponta Do Ouro Mozambique

**Keywords:** animal behaviour, ectoparasites, intra‐specific competition, marine ecology, photo‐ID, predation, *Tursiops aduncus*

## Abstract

Understanding interactions within and between species is crucial to ecological research. However, for cetaceans such interactions can be difficult to observe in the field. Photographs offer an opportunity to study intra‐ and inter‐specific interactions, by capturing ‘snapshots’ of their occurrence over space and time. At‐surface and underwater photographs of bottlenose dolphins (*Tursiops aduncus*) inhabiting Ponta do Ouro Partial Marine Reserve (PPMR), Mozambique, were used to examine evidence of interactions with other dolphins, predators and ectoparasites. Intra‐specific scarring levels significantly differed by sex and age class, with males displaying more scarring than females. Similarly, adults had more scarring than juveniles or calves. Shark bites significantly differed in their distribution across dolphin body areas, with the dorsal side being more frequently wounded than the ventral side. The presence of barnacles was exclusive to fluke, dorsal and pectoral fins, and showed strong seasonal trends. Overall, this study demonstrates the value of photographs for examining marine ecological interactions. It provides the first insights regarding dolphin social behaviour, predation risk and health for this population. These in turn will support future research into the population dynamics and conservation of the PPMR dolphins, which is urgently required in the face of locally increasing anthropogenic pressures.

## Graphical abstract

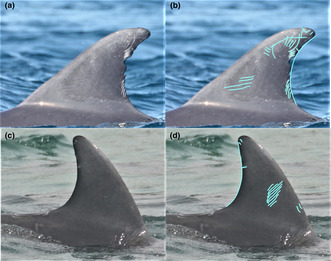

## 1 INTRODUCTION

Understanding intra‐ and inter‐specific interactions is a key aspect of ecology. Interactions with conspecifics form the foundation of social behaviour, which in turn influences social systems and life history strategies (Grueter et al., 2020). The influence of predators drives animal habitat use, grouping behaviours and reproductive strategies (Sheriff et al., 2020). And relationships with parasitic species can impact individual health, behaviour and reproductive success (Lockley et al., 2020). All these interactions go on to drive population dynamics, and thus they are of key importance to conservation and management.

However, studying intra‐ and inter‐specific interactions can be challenging in the case of cetaceans. Firstly, it can be challenging to find and spend significant time with animals due to their fast‐moving and wide‐ranging nature. Field conditions restrict observations to at or near the surface, leaving the majority of interactions undetected. Some interactions can be relatively infrequent and short‐lived, meaning that the likelihood of being ‘in the right place at the right time’ is slim. Finally, in the case of many parasitic species, records are restricted to stranded or bycaught animals that may not be representative of the wider population. Thus, first‐hand, in the field, documented observations of intra‐specific interactions, predation attempts and parasitism involving wild cetaceans are rare.

Many dolphin studies instead infer the occurrence of intra‐ and inter‐specific interactions using photographic data. Scars and natural markings have been used for individual identification of dolphins for decades, leading to extensive photographic databases that allow researchers to study individual animals over long periods of time (Wilson et al., 1999). Whilst these databases are most commonly used for photographic‐identification (photo‐ID), mark‐recapture analyses and population estimates (e.g. Arso Civil et al., 2019; Berrow et al., 2012; Smith et al., 2013), photographs also provide a means to study other aspects of dolphin ecology.

One of the most frequently observed markings on dolphins are tooth‐rake scars. Typically obtained through agonistic interactions with conspecifics, tooth rakes are long, thin, parallel scratches on the dolphin's skin that can take from 5 to 24 months to fade (Lee et al., 2019; Scott et al., 2005; Wilson et al., 1999). Dolphins are known to employ a range of aggressive behaviours in their social interactions (Hamilton et al., 2019; Parsons et al., 2003; Scott et al., 2005). However, aggressive events are difficult to observe in the wild; so studies are increasingly using tooth‐rake scarring as an indirect indicator of intra‐specific aggression. Previous analyses of tooth rake scarring have indicated sex‐specific differences, with male dolphins displaying significantly higher scarring levels than females, likely due to intra‐sexual conflict over access to mating opportunities (Tolley et al., 1995; Rowe & Dawson, 2009; Marley et al., 2013; Orbach et al., 2015; James et al., 2022; Serres et al., 2023). Geographic variations in scarring levels have also been recorded, with variation both within and between populations inferring differences in aggressive interactions due to habitat use, resource availability, age/sex ratios or social structures (Hamilton et al., 2019; Marley et al., 2013; Serres et al., 2023). These findings indicate that intra‐specific aggression has complex individual‐, population‐ and ecosystem‐level drivers.

Another distinctive source of dolphin scarring comes from predation attempts. Sharks represent a key predatory threat to many tropical and sub‐tropical dolphin populations, and shark occurrence is known to influence habitat use and incite anti‐predator behaviours in dolphins (Heithaus & Dill, 2002). However, dolphin predation events are rarely observed, despite studies of stomach contents suggesting that marine mammals comprise a significant proportion of the diets of several large shark species (Heithaus, 2001a, 2001b; Malcolm et al., 2001). Although successful predation events are difficult to quantify, failed attempts can be inferred by the presence of characteristic crescent‐shaped injuries (Castelblanco‐Martínez et al., 2021; Heithaus, 2001b; Melillo‐Sweeting et al., 2022; Nicholls et al., 2023; Smith et al., 2018; Sprogis et al., 2018). Photographic data facilitates documentation of shark‐induced injuries and scars, allowing estimation of predation risk by examining failed attempts (Melillo‐Sweeting et al., 2022). Using this method, past studies have identified variations in shark bites according to dolphin body area and size, foraging strategy, habitat use, group size and species (Castelblanco‐Martínez et al., 2021; Heithaus, 2001b; Melillo‐Sweeting et al., 2022; Smith et al., 2018; Sprogis et al., 2018). Understanding predation risk via photographic analyses provides insight into predator–prey dynamics and drivers of some dolphin behaviours.

Photography provides a non‐invasive means of conducting health assessments of wild dolphins (Thompson & Hammond, 1992; Toms et al., 2020). This technique has been successfully used for pathological and parasitological studies, for example the prevalence of skin lesions (Murdoch et al., 2008), parasitic copepods (Vecchione & Aznar, 2014) and lampreys (Miočić‐Stošić et al., 2020). In some cases, photographic assessment of dolphin populations had similar sensitivity and specificity scores to commonly used screening tests in medicine and veterinary medicine (Murdoch et al., 2008). Semi‐parasitic epizoic organisms can also be quantified from photographic data; although they do not feed upon cetaceans directly, in large numbers such organisms can create substantial drag that imposes a considerable energetic demand on the host, as well as considerable skin damage at attachment sites (Hermosilla et al., 2015). Heavy barnacle infestation in wild dolphins can also be signals of overall poor health, with high loads linked to immunosuppressive effects of viral infection and high contaminant concentrations (Aznar et al., 2005). Thus, photographic data can be used to monitor both direct and indirect indicators of dolphin diseases, which has relevance for individual, population and even ecosystem health.

Analysis of photographic data therefore offers a simple, non‐invasive avenue to examine differences in intra‐ and inter‐specific interactions that may be difficult to directly observe. Results from such studies offer insight into dolphin behaviour, habitat use, reproductive success, and population dynamics, thus adding value to conservation efforts and management decisions. This is particularly true for relatively small, poorly studied dolphin populations exposed to multiple stressors.

Such a population exists within the Ponta do Ouro Partial Marine Reserve (PPMR) in Mozambique. Commercial dolphin tours have been taking place in this area since 1994 and now represent the largest swim‐with‐dolphins industry in the country. One of the tourism operators (Dolphin Encountours Research Centre, DERC) has been collecting photo‐ID and behavioural data since 2007, which has been used to estimate a population size of approximately 300 Indo‐Pacific bottlenose dolphins (*Tursiops aduncus*) utilising the PPMR (unpublished data 2007–2021). DERC data has also contributed towards development of a voluntary code of conduct amid increasing concerns surrounding human disturbance (Rocha et al., 2020, 2022). However, relatively little is known about the ecology of this dolphin population.

This study aims to use photographic data to assess intra‐ and inter‐specific interactions involving the PPMR dolphin population. This is achieved using a combination of dorsal fin images from the photo‐ID catalogue and opportunistic body images taken both at the surface and underwater. Firstly, we evaluated sex‐ and age‐related patterns in intra‐specific aggression by quantifying dorsal fin scarring levels. Secondly, we assessed the frequency and severity of shark‐bite scarring by dolphin body region, sex and age. Finally, we measured the abundance of barnacles in relation to dolphin body region, age and season. Outcomes will provide the first insights into social behaviour, predation risk and health of this dolphin population.

## 2 METHODS

The PPMR stretches for 86 km along the Mozambique coast, from Inhaca Island (−26.0214, 32.9482) in the north to Ponta do Ouro village (−26.8443, 32.8953) near the South African border. With an extension of three nautical miles into the Indian Ocean, the reserve covers approximately 678 km^2^.

Non‐systematic boat‐based surveys were conducted during dolphin tours run by DERC between 2016 and 2018. The operator used an 8 m semi‐rigid inflatable equipped with two four‐stroke, 90 HP engines. Data collection was opportunistic and observation effort varied based on environmental conditions and customer requirements. A typical survey route lasted up to 1.5 h, which involved launching from Ponta do Ouro village and travelling along the coast approximately 500 m from shore, initially southwards (maximum ~1.5 km) then back northwards (maximum ~15 km) until dolphins were encountered.

When dolphins were encountered, data pertaining to location, group composition, predominant activity state and environmental conditions was collected. If conditions were appropriate for approaching the dolphins (i.e. depending on dolphin behaviour, location and weather), photo‐ID and further behavioural data was collected using a group follow protocol (Mann, 1999). Above‐surface images were collected using a Canon ECO 760D with a 70–300‐mm lens focusing on the dorsal fins. Underwater photos were collected using a GoPro H4 focusing on the entire body of the dolphin.

In accordance with PPMR regulations, dolphins were approached for a maximum of 20 min per group. Follows ceased when the dolphin group departed, if an aggressive and/or avoidance behaviour was observed (e.g. tail slap, jaw clap, charge), when weather conditions deteriorated, or when the time limit was reached.

Subsequent individual identification followed standard photo‐ID techniques (see Wilson et al., 1999). Sex of individuals was confirmed by a clear view of the genital region or repeated observations with a calf. Age was determined visually using body size and speckling (Kemper et al., 2019; Krzyszczyk & Mann, 2012; Sprogis et al., 2018; Yagi et al., 2022). If it was considered unclear, then sex and/or age were marked as ‘unknown’.

### 2.1 Dolphin‐inflicted scars

Although dolphin‐inflicted scars can occur on any part of the body, these can be difficult to distinguish underwater and are most reliably viewed via at‐surface photos. Therefore, only dorsal fin images from the DERC photo‐ID catalogue were used to evaluate intra‐specific scarring by dolphin sex and age. This catalogue uses images collected from 1998 to 2018, representing 217 individual dolphins. All of these could be aged (158 adults, 37 juveniles, 22 calves), but sex was only known for adult dolphins (77 males, 81 females).

**FIGURE 1:**
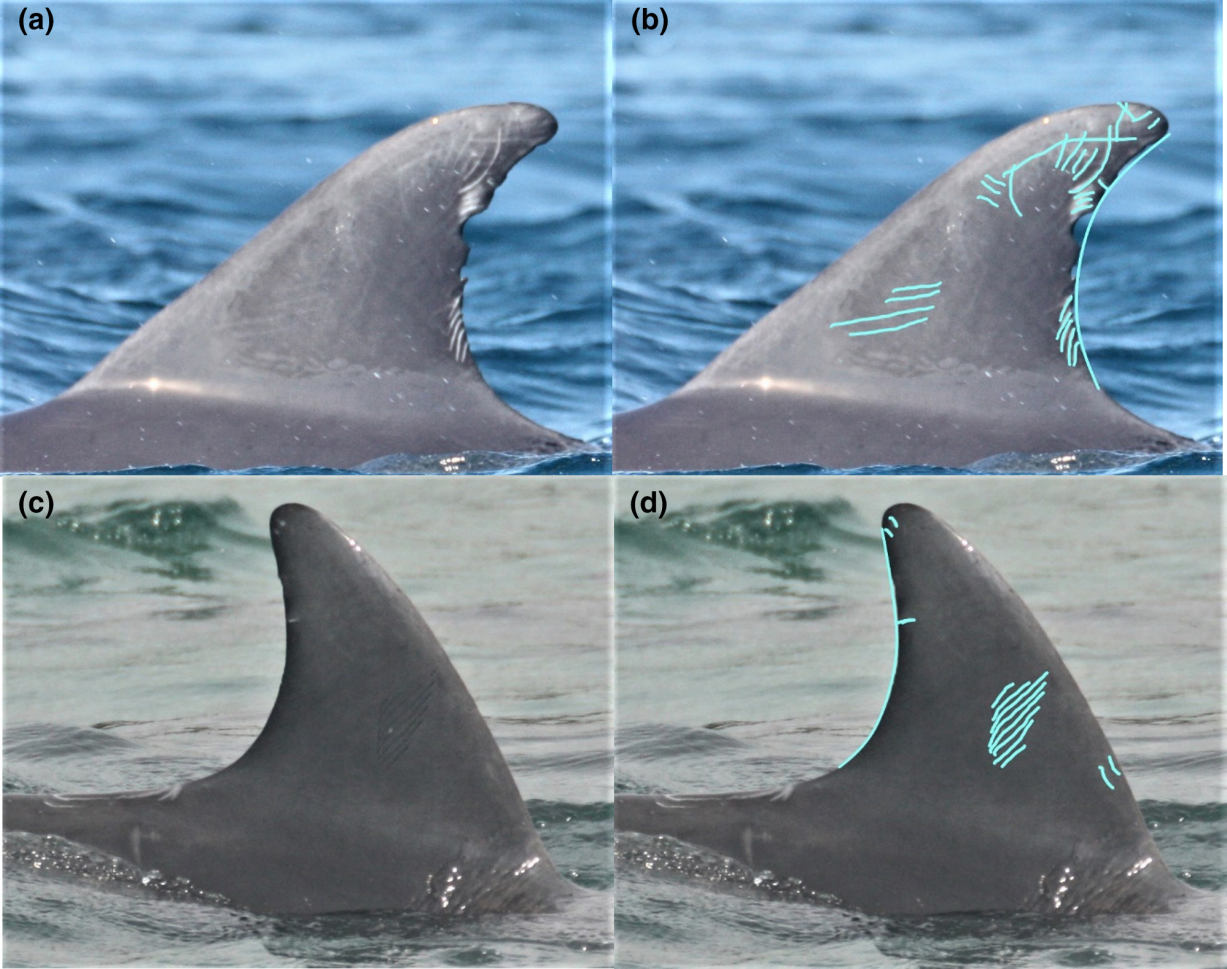
Example of the methods for quantifying intra‐specific scarring, where (a) shows the ID photo of an adult individual before editing and processing, (b) shows the process that was used to work out Rake Direction, Scarring Percentage and Nick Percentage for this individual who was found to have (RD = 6, SP = 45% and NP = 20%). Similarly, (c) is the ID photo of a juvenile individual before editing and processing and image (d) shows the process used to work out the scarring data for this individual (RD = 3, SP = 20% and NP = 5%).

Three techniques of quantifying scarring were applied to each individual, following the methods of Marley et al. (2013). Firstly, *Rake Direction* (*RD*) was calculated by counting the number of tooth rake directions on the dorsal fin of each individual; if an individual had an image for both the left and right sides of their dorsal fin, the average of both sides was taken. Directions were defined as tooth rake scars that ran in different directions to each other, as these were assumed to represent different aggressive events. For example, four parallel tooth rakes running vertically, two rakes running horizontally and seven rakes running obliquely were considered as a total of three directions. *Scarring Percentage* (*SP*) was a visual estimate of the proportion of the dorsal fin covered by tooth rake scars, which again was averaged if images were available of the left and right side of the same individual. Finally, *Nick Percentage* (*NP*) was a visual estimate of the proportion of the trailing edge of the dorsal fin missing due to nicks and tears. The pen tool in Windows 10 was used to aid visualisation of scarring (Figure 1).

### 2.2 Shark‐inflicted scars

Underwater photographs taken between 2016 and 2018 were reviewed for evidence of shark‐bite wounds. If present, the sex and age of the dolphin were recorded (if known), along with details on bite position and severity. Multiple underwater photographs of each individual were collected during each encounter, facilitating high probability of detecting a shark‐inflicted scar, if present.

The position of the bite on the dolphin's body was recorded as a specific area and then broadly grouped into two body regions: dorsal or ventral. The former included the head, anterior, mid‐flank, dorsal fin, anterior peduncle and posterior peduncle; the latter included the throat, chest, pectoral fins, belly and ventral peduncle. Note that jaws and flukes were excluded from the body regions due to ambiguity regarding directional origin of the predation attempt. If a bite was present over multiple areas, then the area containing the majority of the wound was considered as the targeted area, unless there was substantial scarring across all areas.

**FIGURE 2:**
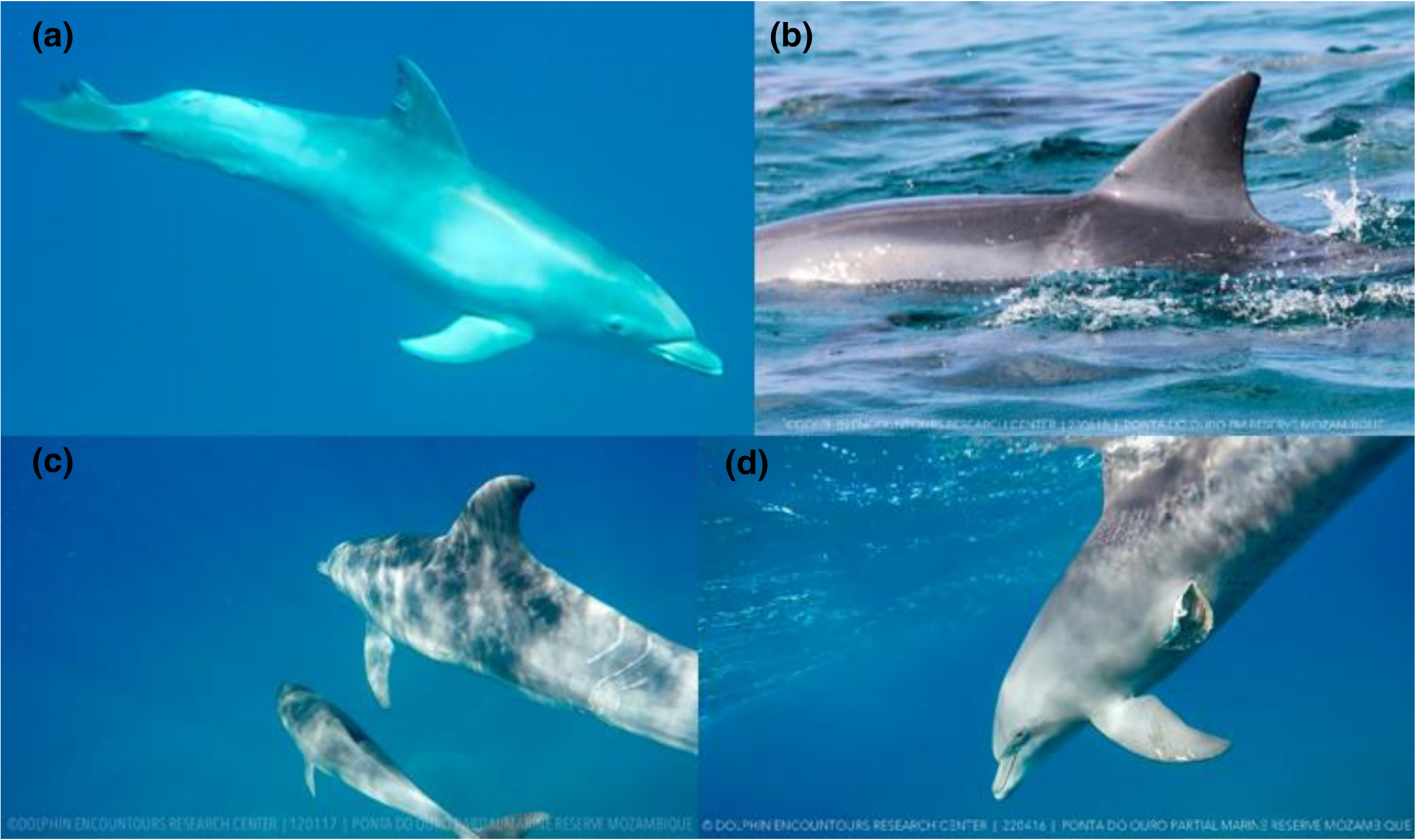
Example of the methods for ranking the severity of shark bites, which was qualitatively scored on an ordinal scale from (a) 1 healed wound to (d) 4 open wound.

As the photos were taken after the event, it was not possible to know the exact date the predation attempt occurred. Therefore, the severity of the wound was qualitatively scored on a scale (from 1 [healed wound] to 4 [open wound]; see Figure 2).

### 2.3 Ectoparasites

**FIGURE 3:**
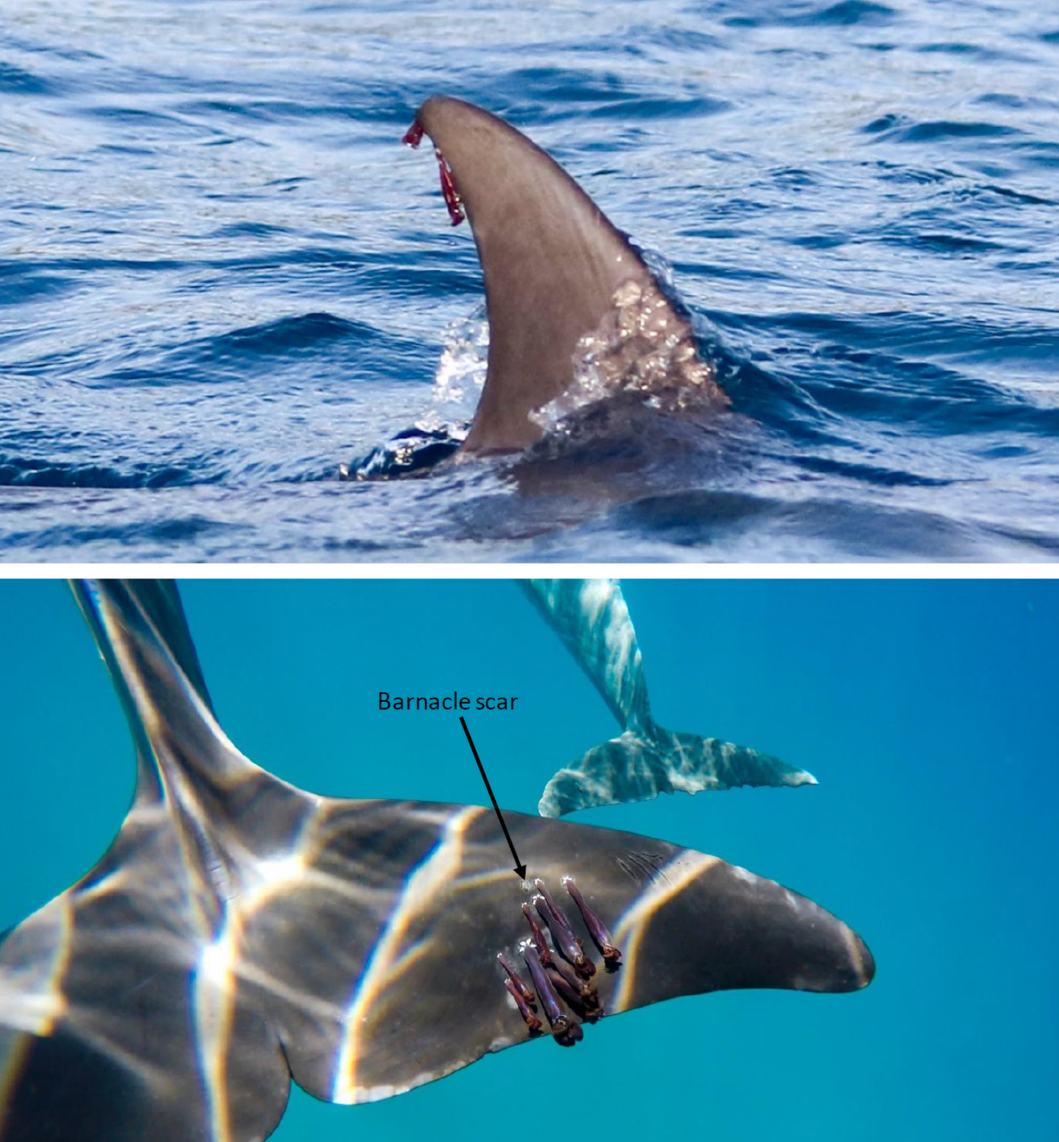
Examples of barnacles observed on the dorsal fin and flukes of dolphins.

At‐surface and underwater images taken throughout 2018 were reviewed for evidence of ectoparasites; however, the only ectoparasites observed were barnacles (Figure 3). When ectoparasites were present, the sex and age of the dolphin were recorded (if known), along with details on the infected body area and the maximum discernible number of parasites.

Additionally, the month in which the photo was taken was recorded to facilitate investigation of temporal trends. This was further investigated using mean monthly sea surface temperature (SST) data, which was derived for the study site using MODIS‐AQUA rasters analysed in QGIS.

### 2.4 Data analysis

Statistical analyses were conducted in SPSS (vr 27), Minitab (vr 19) and R (vr 4.2.0) using a significance threshold of 0.05. Due to the nature of the data (i.e. response variables based on frequencies, proportional or ordinal data), non‐parametric tests were employed. Dolphin‐inflicted scarring (RD, SP and NP) were compared between sexes using Mann–Whitney tests, whilst age classes were compared using Kruskal–Wallis tests and post‐hoc Mann–Whitney tests with Bonferroni corrections. Shark‐inflicted scars were compared by body region in terms of frequency and severity, respectively, using chi‐square tests and Kruskal–Wallis tests. Ectoparasite presence was compared by body region and dolphin age using chi‐square tests, and the association between ectoparasite counts and SST was investigated using Spearman's rank correlation.

## 3 RESULTS

### 3.1 Sex‐ and age‐specific patterns in dolphin‐inflicted scars

**FIGURE 4:**
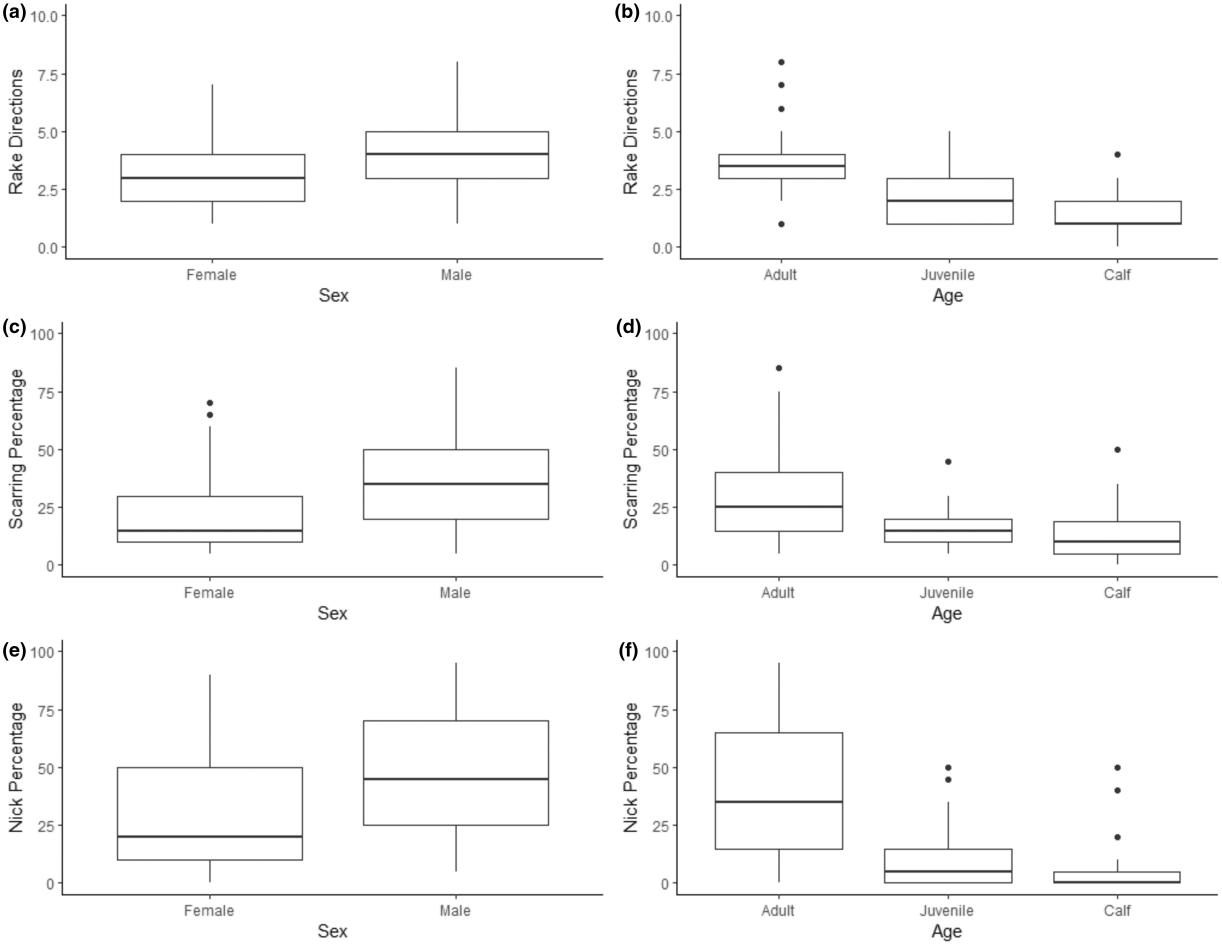
Intra‐specific scarring by sex and age of individual dolphins.

Of the 217 individual dolphins examined, all showed evidence of dolphin‐inflicted scarring with the exception of three calves. All three techniques of quantifying intra‐specific scarring indicated a significant difference in scarring levels between sexes and age categories (Figure 4).

Male dolphins (*n* = 77) showed significantly higher levels of scarring than females (*n* = 81) in terms of rake direction (*U* = −4.798, *p* < .001), scarring percentage (*U* = −5.158, *p* < .001) and nick percentage (*U* = −3.740, *p* < .001). For males, median values were 4.0 (RD), 32.5% (SP) and 47.5% (NP); for females, median values were 3.0 (RD), 15.0% (SP) and 20.0% (NP).

Adults (*n* = 158) showed significantly higher levels of scarring than juveniles (*n* = 37) and calves (*n* = 22) in terms of rake direction (*H* = 44.15, *p* < .001), scarring percentage (*H* = 27.22, *p* < .001) and nick percentage (*H* = 74.68, *p* < .001). Post‐hoc tests indicated that in all cases, adults were significantly different to both juveniles and calves, whilst the latter two age categories did not significantly differ from each other. Adults had median values of 3.5 (RD), 25.0% (SP), and 35.0% (NP); juveniles 2.0 (RD), 15.0% (SP) and 5.0% (NP); and calves 1.0 (RD), 10.0% (SP) and 0.0% (NP).

### 3.2 Frequency and severity of shark‐inflicted scars

A total of 21 shark‐inflicted scars were recorded on 14 individual dolphins, representing 4.7% of the estimated population. These included one male, seven females and six of unknown sex. The majority of individuals (57.1%; *n* = 8) had a single scar, followed by two scars (7.1%; *n* = 5), and a single adult female had three scars.

**FIGURE 5:**
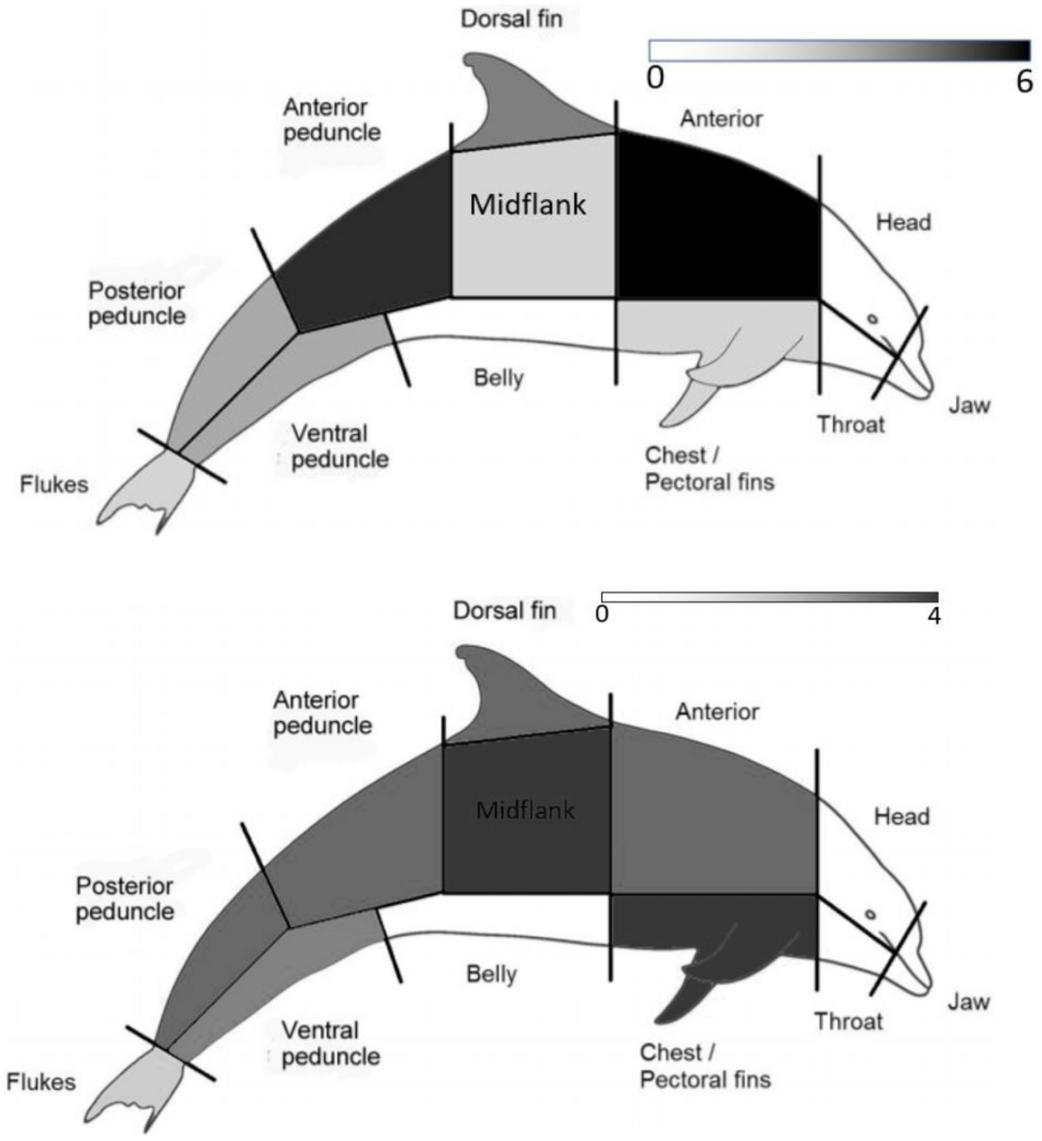
Heatmaps of (a) scar frequency and (b) severity by dolphin body area.

The frequency of shark‐inflicted scars significantly differed by body region (X^2^ = 9.8, *p* < .001) (Figure 5). In general, the dorsal region (85.0%; *n* = 17) was more frequently inflicted with shark scars than the ventral region (15.0%; *n* = 3). The most scarred body areas were the anterior (28.6%; *n* = 6), anterior peduncle (23.8%; *n* = 5), dorsal fin (14.3%; *n* = 3), posterior peduncle (9.5%; *n* = 2) and ventral peduncle (9.5%; *n* = 2). The chest, mid‐flank and fluke areas each had one scar. No shark‐inflicted scars were observed on the head, jaw, throat or belly.

Only 23.8% (*n* = 5) of injuries were fresh, open wounds (i.e. severity score of 4), with the majority (76.2%; *n* = 17) in various stages of healing (i.e. severity scores from 3 to 1). The severity of shark‐inflicted scars showed no significant difference by body region (*H* = 0.030, df = 1, *p* = .954) (Figure 5).

Due to the low sample size, no further analyses were conducted by sex. Calves and juveniles were combined to form a ‘non‐adult’ category (*n* = 6) for comparison with adult dolphins (*n* = 7). However, there was no significant difference between adults and non‐adults in terms of scarring frequency (X^2^ = 0.077, *p* > .05) or severity (*H* = 1.491, df = 1, *p* = .222).

### 3.3 Prevalence and seasonality of barnacles

Throughout 2018, a total of 35 dolphins were observed with signs of barnacles, representing approximately 11.7% of the estimated dolphin population. Of these, 33 had barnacles attached, one had barnacles attached and evidence of barnacle scarring, and the remaining individual contained prominent barnacle scars on their fluke. Subsequent analyses only considered dolphins who currently had barnacles attached rather than evidence of previous infestation.

A total of 118 barnacles were counted, with a maximum of 12 barnacles on an individual infected dolphin. Barnacles were only observed on dolphin extremities, predominantly the fluke (77.1%; *n* = 91), followed by the dorsal (11.9%; *n* = 14), and pectoral fins (11.0%; *n* = 13).

**FIGURE 6:**
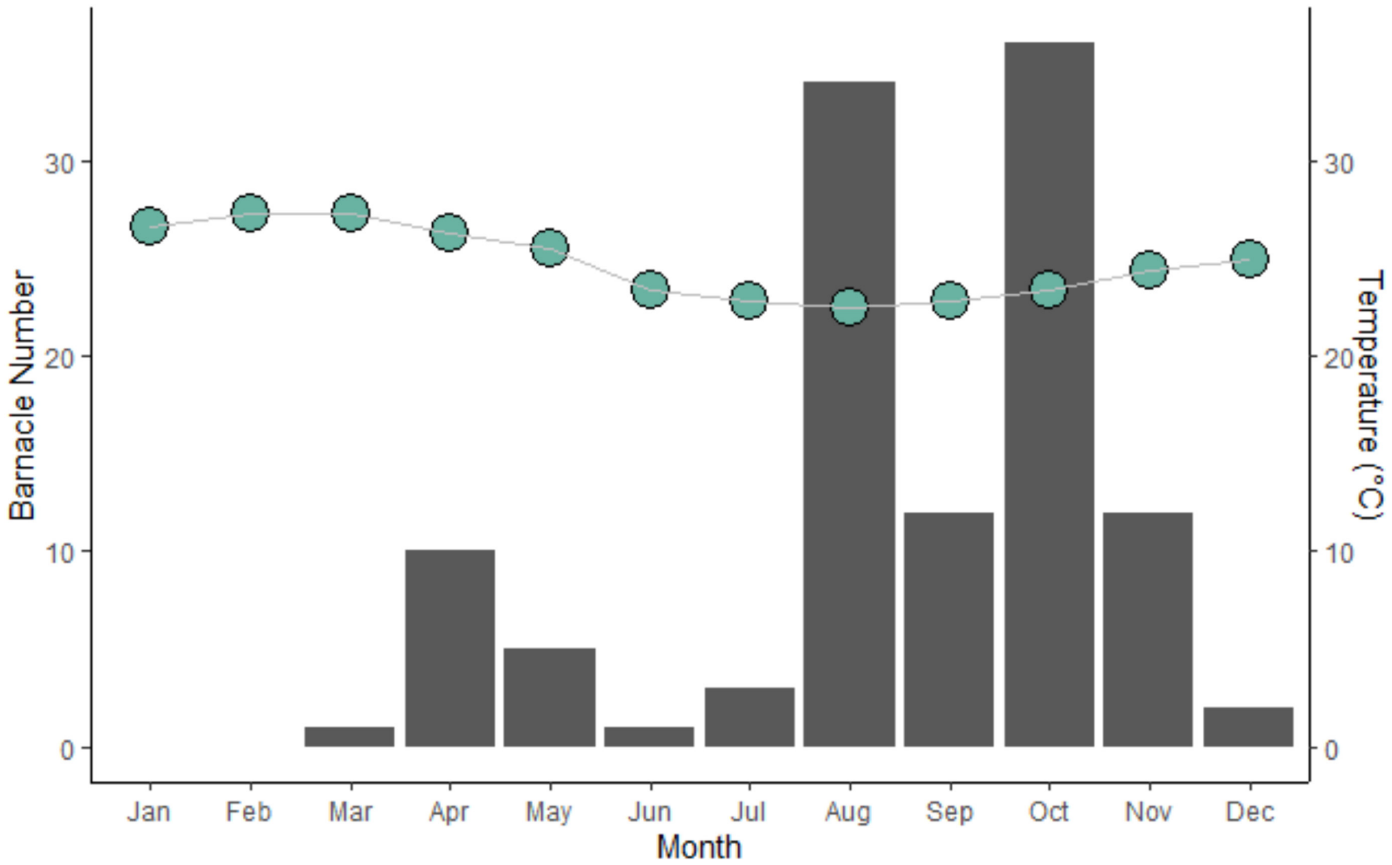
Patterns of barnacle occurrence (bars) and mean monthly sea surface temperature (dots) by month.

Barnacle infestation rate changed over the course of the year (Figure 6). No barnacles were observed in January or February, but then infestation rates gradually increased from March to July before peaking from August to October, and finally declining again across November and December. This corresponded with mean monthly SST, which showed a significant negative correlation with barnacle counts (*r*_s_ = −0.657, *p* < .05; Figure 6). Throughout 2018, mean SST ranged from 22.5°C to 27.3°C, with the warmest months (January to March) having the lowest barnacle counts and the coolest months (August and October) having the highest barnacle counts.

## 4 DISCUSSION

In this study, we demonstrated the usefulness of photographic data for assessing the intra‐ and inter‐specific interactions of dolphins. Dorsal fin images revealed sex‐ and age‐specific differences in terms of surface features and edge marks, indicating differential interactions with conspecifics. Whole‐body images captured evidence of past predation attempts that differed between dolphin body regions. Whole‐body images also documented parasitic infestations, which were prevalent in particular body areas and displayed seasonality in occurrence. As these interactions are typically difficult to observe in the field for marine mammals, photographs offer an opportunity to act as indicators of conspecific behaviour, predation and dolphin health.

### 4.1 Dolphin‐inflicted scars

Sex‐specific differences in dolphin‐inflicted scars most likely reflect differing behaviours between males and females. Males were more heavily scarred than females, according to all three metrics used. On average, male dolphins displayed tooth rake scars across approximately one‐third of their dorsal fin with almost one‐half of the fin's trailing edge missing due to nicks, which was more than 2x higher than in females. This aligns with other studies, where male dolphins have also been found to be the more heavily scarred sex, albeit with varying intensities of scarring between populations (Marley et al., 2013; Rowe & Dawson, 2009). Males have previously been found to experience the greatest change in scarring levels over short periods of time, whereas females experienced less pronounced changes (James et al., 2022). Consequently, there have recently been efforts to predict dolphin sex by modelling rate of change in dorsal fin marks, with extremely accurate outcomes (James et al., 2022; Rowe & Dawson, 2009). The reason behind these differences likely relates to differences in social behaviour, particularly mating behaviours. Previous studies have described female–female aggression as rare to non‐existent among bottlenose dolphins, whereas aggression by males towards females and other males is considerably higher (Mann & Smuts, 1999; Samuels & Gifford, 1997; Scott et al., 2005; Tolley et al., 1995). Although the mating system of the PPMR dolphin population has not been exclusively investigated, numerous studies of other Indo‐Pacific bottlenose dolphin populations have revealed the existence of male alliances that work together to herd and sexually coerce females, even ‘stealing’ females from other alliances (Connor & Krützen, 2015; Hamilton et al., 2019; Möller et al., 2001). Consequently, cycling females are significantly more likely to have fresh tooth rakes than other females (Scott et al., 2005). This suggests that female dolphins mostly receive scars from males during mating attempts, whilst males likely receive scars as a result of intra‐sexual competition over access to females. Recent studies have also identified ecological variation in alliance‐related aggression among male bottlenose dolphins, indicating more intense competition in some habitats (Hamilton et al., 2019). It would be beneficial in future studies to review associations between individual dolphins to investigate the presence of male alliances and potential breeding strategies within the PPMR population. However, recent studies also suggest that female dolphins may heal faster than males (Lee et al., 2019); thus, further investigations into scar accumulation and healing times within the PPMR dolphins would also be beneficial to confirm whether higher scarring rates are genuine.

This link between aggressive interactions and mating opportunities may also partially explain the age‐specific differences in dolphin‐inflicted scars observed in the present study. Very little scarring was seen among juveniles or calves, whereas adults had significantly higher scarring levels. Although it could be argued that scarring may just accumulate over time, this does not apply to all three metrics. Dorsal fin nicks represent tissue loss and whilst nick shape can alter over time as further tissue is lost, it rarely regrows (though see how images of nicks can be misleading in Quick et al., 2017). In comparison, tooth rake scars are non‐permanent, lasting up to two years (Lee et al., 2019; Scott et al., 2005). Therefore, whilst a higher nick percentage metric might be expected of older animals due to greater time for accumulating permanent markings, the non‐permanent markings measured by the rake direction and scarring percentage metrics should not be dissimilar between age groups. Indeed, permanent markings were 7x higher in adults than juveniles, whilst non‐permanent markings in adults were 1.7x higher than juveniles and 2.5x than calves. The fact that a significant difference exists between adults and non‐adults across all metrics implies that this transition to adulthood brings with it a behavioural change that increases the likelihood of scar accumulation. Sexual maturation and associated agonistic interactions are likely explanations. Conversely, scarring studies in Shark Bay found higher tooth rake prevalence among juveniles than adults, with a dip in scarring levels as animals reached sexual maturity (Lee et al., 2019). This may reflect stable male–male bonds resulting from alliance formation; thus, further information regarding the mating strategies of PPMR dolphins could help explain observed scarring patterns and inferred aggressive behaviour.

The present study was only able to confirm the sex of adult animals, but other studies have been able to investigate sex‐ and age‐specific differences in more detail. Juvenile males in Shark Bay were noted to have the greatest presence of tooth rakes across all age and sex categories, engaged in playful and sexual practice behaviours with each other that subsequently turned aggressive, and frequently directed aggression towards adult females (Scott et al., 2005). Male calves in the Shark Bay population were also significantly more aggressive than female calves (Scott et al., 2005). It would be interesting to take a more retrospective look at the PPMR photographic records to investigate whether imagery exists for sexed adult dolphins when they were in the juvenile or calf stage, then examine when scarring levels begin to increase, at what point sex differences in aggressive behaviour emerge, and if any seasonality in scarring occurs.

### 4.2 Shark‐inflicted scars

Overall, relatively few of the PPMR dolphins (~5%) displayed shark‐inflicted scars compared to other bottlenose dolphin populations, such as: Shark Bay, Australia (74%; Heithaus, 2001b); Sarasota, Florida (36%; Wilkinson et al., 2017); Bimini, the Bahamas (29%; Melillo‐Sweeting et al., 2022); northwest Australia (18%; Smith et al., 2018) and southwest Australia (17%; Sprogis et al., 2018). However, the number of individuals with shark‐inflicted scars should be considered a minimum estimate, given the non‐systematic sampling design, differential sighting rates for individuals and the potential for old, well‐healed injuries to be missed (Melillo‐Sweeting et al., 2022).

When they did exist, shark‐inflicted scars predominantly occurred on the dorsal region. This aligns with other studies that have also documented more frequent shark bite scars on the dorsal region of dolphins (Melillo‐Sweeting et al., 2022). This is particularly interesting given that other studies were often limited to viewing at‐surface images with only sporadic views of the ventral region, whereas underwater photography allowed us to view the body in full. However, it should be remembered that such scars represent failed predation attempts; it can be assumed that shark attacks targeting the softer, ventral side of dolphins (e.g. throat, belly) are likely to have a greater success rate (Heithaus, 2001b; Melillo‐Sweeting et al., 2014). Indeed, some shark species are known to attack by rushing vertically from depth to ambush prey (Martin et al., 2009; Martin & Hammerschlag, 2012). Similarly, no shark‐inflicted scars were recorded on the head or jaws, which again would likely be fatal for the dolphin. Although there were no age‐specific differences in the frequency of shark‐inflicted scars, it may be that dolphin calves are less likely to survive shark attacks due to their smaller size. Some studies suggest that sharks may specifically target young dolphins when hunting, which in turn may drive calving seasonality (Fearnbach et al., 2011). The current study could not investigate sex‐specific differences in shark‐inflicted scars due to low sample size, with only eight sexed individuals bearing evidence of shark‐inflicted scars. However, of these, a disproportionate number were female (seven versus one male). This is in contrast to other studies, where adult males bore more shark scars than adult females (Heithaus, 2001b). It would be useful to continue recording evidence of predation attempts in the PPMR dolphins in the hope of improving the sample size of sexed dolphins, as this may facilitate future investigations into sex‐specific differences in predation risk responsiveness and avoidance.

Several large shark species are known to occur within Mozambique waters, some of which are known to predate upon dolphins (Daly, 2018). Telemetry studies have shown that tiger sharks (*Galeocerdo cuvier*) tagged in the PPMR move between coastal waters, the continental shelf and offshore reef systems, with some even crossing the Mozambique Channel towards Madagascar (Daly et al., 2018). Tiger sharks are generalist predators that forage in a variety of habitats. Studies on diet and trophic ecology of tiger sharks caught in the neighbouring waters of the KwaZulu‐Natal coast in South Africa have shown these animals to consume a broad spectrum of prey items, most commonly other elasmobranchs (55% frequency of occurrence), teleosts (51%), mammals (41%), birds (27%), cephalopods (16%), crustaceans (13%) and reptiles (6%) (Dicken et al., 2017). The type of mammal typically consumed changed with shark size; small odontocetes (including bottlenose dolphins) were the most commonly consumed prey of small and medium tiger sharks, but as shark body size increased then mysticetes (e.g. humpback whales, *Megaptera novaeangliae*) formed a greater proportion of the diet. There was also evidence of seasonal prey switching, with medium‐sized sharks predominantly preying upon elasmobranchs in summer and autumn, whereas mammals became the dominant dietary component in winter and spring, which coincided with the humpback whale migration. However, it was not clear whether prey items consumed were the result of predation or scavenging. In comparison, bull sharks (*Carcharhinus leucas*) tagged within the PPMR exhibit prolonged periods of residency in this area during the austral summer, with many undertaking inshore forays (Daly et al., 2014). However, tagged bull sharks also interspersed their periods of residency with substantial return migration events towards lower latitudes in the austral spring and winter (Daly et al., 2014). Stable isotope analysis demonstrated that adult bull sharks within the PPMR utilise a more diverse habitat range than sub‐adults and consequently consume a greater proportion of larger prey from higher tropic levels (Daly et al., 2013). Stomach content analysis of bull sharks caught in South Africa revealed teleosts and elasmobranchs to be the most common prey group (55% and 50% of stomachs containing food), with mammals (9%) being a more minor prey group (Cliff & Dudley, 1991). Scavenging appeared to be an important contributor to bull shark diets.

The relatively low frequency of shark‐inflicted scars on PPMR dolphins, along with the wide‐ranging movements and diverse diets of sharks, may indicate that dolphins are not a primary food source for sharks foraging in this area – or conversely that shark predation attempts are extremely successful and consequently leave little evidence. It was beyond the scope of this study to try and identify the shark species responsible for the observed scars, but it would be useful to attempt this in the future alongside further dedicated studies of shark ecology and diet. Understanding shark movement patterns and dietary preferences is important because predation risk has been shown to influence dolphin habitat use. In Shark Bay, Western Australia, shallow habitats are more productive than deeper ones, and thus are preferentially used by foraging dolphins – but only when tiger sharks are absent (Heithaus & Dill, 2002). When tiger sharks are present, dolphins trade off food availability with predation risk and spend more time in the relatively safer deep‐water areas. This even influences habitat use at the fine scale, with dolphins choosing to forage in shallow ‘edge’ habitats when sharks are present, likely due to the higher escape potential of these areas and their lower intrinsic risk (Heithaus & Dill, 2006). It would therefore be interesting to further investigate the seasonality of shark predation and potential influence on dolphin habitat choice and space use.

### 4.3 Barnacle infestations

Several sessile barnacle species have been linked to marine vertebrates, with *Xenobalanus globicipitis* specialised for living on cetaceans and reported on 30 species worldwide (Kane et al., 2008). The barnacles observed in this present study are assumed to be *Xenobalanus* based on their general morphology. Previous studies have most commonly observed *Xenobalanus* on the trailing edges of the dorsal fins, pectoral fins and tail fluke (Carrillo et al., 2015; Kane et al., 2008; Moreno‐Colom et al., 2020; Orams & Schuetze, 1998), although these studies have primarily utilised surface‐only photographs (where not all barnacles present on the animals may be visible) or strandings data (where barnacle prevalence may be higher due to poor host health). Our study had the benefit of using both at‐surface and underwater photographs to view the full dolphin body, lending greater confidence to the spatial distribution of barnacles indeed being focused on dolphin extremities.

As in other studies, the body area most predominantly infected was the fluke. This likely reflects optimal feeding opportunities created by eddies shed by the flukes as the dolphin swims (Moreno‐Colom et al., 2020), which may also explain why this body area also typically harbours the largest barnacles (Carrillo et al., 2015). Although the fine‐scale spatial distribution of barnacles within body areas was not investigated in the present study, others have reported greater barnacle abundance on the central third and dorsal side of the flukes, with up to 12x greater barnacle abundance compared to the ventral side (Carrillo et al., 2015; Moreno‐Colom et al., 2020). This appears to be linked with host swimming performance; tail downstrokes are more forceful than upstrokes and the resulting flow patterns may influence the likelihood of contact and/or attachment of barnacles (Moreno‐Colom et al., 2020). Such differences in swimming performance may also explain why barnacles appear to be more prevalent on younger (i.e. slower swimming) dolphins at some sites (Orams & Schuetze, 1998). Similarly, high barnacle abundance has also been reported for sick animals, with up to 100 barnacles reported on a single‐stranded individual although this may also be linked with changes to skin permeability as a result of immunosuppression (Aznar et al., 2005).

Barnacle abundance has also been linked to host habitat‐use. Sperm whales (*Physeter macrocephalus*) and beaked whales (*Mesoplodon* spp.) have not been reported as hosts, which may be linked to the deep feeding depths of these species and the consequential impact on barnacle settlement (Kane et al., 2008). Higher barnacle abundance has been reported in coastal areas versus pelagic waters (Kane et al., 2008; Toth‐Brown & Hohn, 2007; Van Waerebeek et al., 1993), possibly as a result of differing nutrient levels or other oceanographic features. This has been used to differentiate inter‐mixing stocks of inshore and offshore bottlenose dolphins, with the latter having 8x higher average infestation indices than dolphins utilising inshore, estuarine areas (Urian et al., 2018), likely reflecting poor tolerance of brackish waters by the barnacles.

Barnacle occurrence also displays strong seasonal patterns in association with changing water temperatures. Studies in temperate waters have reported highest barnacle abundance in summer months (~25°C; Urian et al., 2018), whereas tropical areas report barnacles to be virtually non‐existent in their warmest months (Orams & Schuetze, 1998). The present study supports this trend, with peak barnacle abundance in the cooler months (~23°C). This may reflect thermal tolerance of barnacles, with *Xenobalanus* thought to be a warm‐water species during the larval phase (Kane et al., 2008). Although adult *Xenobalanus* have been observed in cold waters, this has been on migratory host species who likely obtained the barnacle during time spent in higher latitudes; it has not been observed on cetacean species restricted to polar or cold temperate waters (Kane et al., 2008; Matthews et al., 2020). It is unclear whether seasonal changes in *Xenobalanus* abundance are due to environmental conditions, differential spawning patterns, or if this reflects a lifespan of ~6 months. It should also be noted that surface‐active behaviours such as breaching have been shown to dislodge barnacles (Félix et al., 2006), which could also account for the sudden dips in barnacle numbers following abundant months as seen here. It would be beneficial to examine the conditions associated with barnacle abundance more closely, considering environmental variables (e.g. temperature, salinity, nutrient availability) alongside data on host sex, age, health status and habitat use.

### 4.4 Conclusion

In summary, this study successfully utilised photographic data to provide the first insights regarding the social behaviour, predation risk and health status of the PPMR dolphin population. This paves the way for future research investigating dolphin behaviour, mating strategies and habitat use, as well as aspects of shark diet and barnacle physiology. Understanding interactions within and between species is a key facet of ecology, particularly for small, poorly; Möller studied populations.

## AUTHOR CONTRIBUTIONS

**Sarah A. Marley:** Conceptualization (lead); formal analysis (equal); investigation (equal); methodology (equal); supervision (lead); visualization (equal); writing – original draft (lead). **Laura McConnell:** Formal analysis (equal); investigation (equal); methodology (equal); visualization (equal); writing – review and editing (equal). **Chloe Allen:** Formal analysis (equal); investigation (equal); methodology (equal); visualization (equal); writing – review and editing (equal). **Shaye Wettner:** Formal analysis (equal); investigation (equal); methodology (equal); visualization (equal); writing – review and editing (equal). **Thomas Hunt:** Formal analysis (equal); investigation (equal); methodology (equal); visualization (equal); writing – review and editing (equal). **Diana Rocha:** Data curation (equal); methodology (equal); supervision (supporting); writing – review and editing (equal). **Angie Gullan:** Data curation (equal); methodology (equal); writing – review and editing (equal).

## FUNDING INFORMATION

This was an unfunded project.

## CONFLICT OF INTEREST STATEMENT

The authors declare no competing financial or nonfinancial interests that are directly or indirectly related to this work.

### OPEN RESEARCH BADGES

This article has earned an Open Data badge for making publicly available the digitally‐shareable data necessary to reproduce the reported results. The data is available at http://doi.org/10.6084/m9.figshare.26014522.

## Data Availability

The data used in this project is freely available at http://doi.org/10.6084/m9.figshare.26014522.
